# Hospital Finance

**Published:** 1922-04

**Authors:** 


### HOSPITAL FINANCE.

### THE WILFRED ALLEN SCHEME.

THE steady progress made by the Hospitals' Welfare Society for Lewisham, Deptford and Greenwich, which by a penny-a-week collection from householders in these districts has raised some £6,000 for the Miller, Seamen's, South-Eastern, Blackheath and other hospitals, is proving infectious. Mr. George Harvey, the Mayor of Holborn, is trying to establish a similar scheme for the benefit of the London Homœopathic, the Great Ormond Street, the Italian, St. Paul's and the National Hospitals. The appointment of ward or district committees is relatively easy. The difficulty is to find sufficient voluntary workers to call at the houses to collect the subscriptions, even though, if the streets be intelligently allotted, each collector need give no more time than an hour or two a week. For a similar scheme at Lowestoft, for instance, there are some 200 collectors. Most of the collectors are women, who are less likely than men to be turned away without a word, and the result of the efforts was in 1921 to raise £3,600 in nine months. The scheme then is worth, say, £5,000 a year at present, and all similar schemes grow when once they have been securely started. Why has their development been so long delayed ? It is, literally, for years that we have advocated organised contributions from all sections of society in London on the lines made familiar by the workers' weekly gifts in the industrial north. But the popular belief that they were inapplicable to London was too much for our advocacy. We attribute the very remarkable change to the urgent need for a new and united effort, and to the widespread advertisement which the voluntary hospitals received in consequence of the financial crisis made public two years ago and ever since emphasised in the newspapers. Another remarkable fact is that the household and not the shop or office is providing the easiest unit from which to start. It should be added that the above scheme is often described as the Wilfred Allen Scheme. Mr. Allen is the enterprising chairman of the Hospitals' Welfare Society.

At a meeting at the Guildhall on March 1 (Mr. Alderman E. C. Moore in the chair) Mr. Wilfred Allen explained his scheme to an audience which included several metropolitan mayors and representatives of over 40 hospitals. He said that during the past twelve months they had been able to divide £5,200 among their five local hospitals. The amount collected had risen from £1 19s. 4d. in January, 1920 to £245 in January, 1921 and £402 in January, 1922 ; expenses were met by the proceeds of whist drives, dances and fairs. A weekly house-to-house collection of a penny or more per house was made, regularity being essential to success. The three boroughs, which contained 75,000 houses, were divided into ten areas, each presided over by a divisional superintendent. Each division contained five sections, and each section eight units. Each unit supervisor had from eight to twelve collectors under her, and from 20 to 30 houses were allotted to each collector, who was furnished with a box of distinctive shape and a card as her authority. The unit supervisors called for their collectors' money each week and handed it on to the section supervisors. These in turn were visited by the divisional superintendents, who paid the money into the bank, no part of it passing through the office of the society. At least 1,700 ladies were now engaged in the work.

The Society has recently accepted an invitation from the local hospitals to organise mass collections from workshops, factories and shops.

### THE CAPITALISATION OF ANNUAL SUBSCRIPTIONS.

WE should like to know what success has attended a scheme adopted by the board of the Saffron Walden Hospital for the capitalisation of annual subscriptions. Its author is Mr. F. S. H. Judd, the hospital's honorary treasurer. By this scheme a subscriber commutes his annual subscription by transferring to the board a capital sum. The income from this is credited as his annual subscription during his life. The idea is that " as hospitals pay no income tax, the subscriber would benefit to the amount of the current tax," and those intending to bequeath legacies to the hospital would relieve their estates from legacy duty, and in certain cases from estate duty, by an immediate gift in regard to the sum of their commuted annual subscription. The scheme as such has obvious advantages, but does it not presuppose an ability on the part of annual subscribers which only a few of them possess ? How many of our ordinary hospital's annual subscribers of say £10 feel that they can afford a present gift of £250 ? This is a question of fact and can be easily settled. We hope, therefore, that Mr. Judd will let us know how far this ingenious scheme has proved successful.

### MUNICIPAL CONTRIBUTIONS TO HOSPITALS.

THE Parliamentary Committee of the Sheffield Corporation has reported that only by Act of Parliament will the City Council be able voluntarily to contribute to the sums given by its staff to the voluntary hospitals. Thus, the Corporation has no legal power to add an employer's contribution to their gifts. The Town Clerk, however, has an ingenious notion whereby the legal difficulty might be overcome. He has expressed the opinion that if the terms of agreement with the Corporation's employees were varied so as to provide for a voluntary deduction of one penny in the £ for the Sheffield hospitals, then the contract of service could lay down that the Corporation should add to the deducted sum an amount equal to one-third of its value. We note the suggestion in case it may prove practical and an example. But if the Corporation has no legal powers to add voluntary gifts to its employees' contributions to hospitals, how has it legal power to contract with them to do so ? The distinction seems subtle. Is it valid ?

### RATES FOR CHARITY.

THE most far-reaching suggestion for linking Municipalities with voluntary hospitals was lately made by a speaker at Liverpool. He proposed a scheme whereby subscriptions for charities should be made by the rates through the Municipality, which should distribute the total, and thus, while leaving the charities free from interference, spread the support of charity over all the citizens. This is the organisation of charity with a vengeance, and, in practice, would mean municipalisation without disguise. Once people are rated for hospitals, the rate will be indistinguishable from any other, and, in consequence, the cost of administration will increase, and the medical staffs expect to be paid. The novel feature of the proposal is that it was put forward by an advocate of the voluntary system. But it shows that organisation can be advocated to such lengths as to become indistinguishable from a municipal system.

---

### SIR W. DIAMOND'S SCHEME.

A less drastic and more inviting proposal was lately made by Sir William Diamond, chairman of King Edward VII.'s Hospital, Cardiff, when the guest of the Lord Mayor. Remarking that the maintenance of the hospital was as much the Corporation's interest as anyone's, he invited "the co-operation of the representatives of the various wards on the City Council in setting up the necessary machinery to induce residents to subscribe regularly." Private persons who have voluntarily undertaken the house to house collection of weekly sums have had such success that the plan must be developed, but for the hospital committee to form the necessary machinery would be a very heavy task. The organisation in the various wards of the city was in existence and could, if his hosts agreed, be used without difficulty. Sir William added that in rates and taxes the hospital paid £320, for water £540, for electric light £1,235, a total of over £2,000, against which the annual contribution of the City Council was £500. During the past year the cost per head had been reduced by £16.

It is satisfactory to know that the changes made during the past year have been successful. A medical superintendent has been appointed, which leaves Mr. Leonard Rea free to devote himself to finance. The income has increased by £7,000, and the expenses have been reduced by over £4,000. This is a notable feat. The next step is to secure a charter of incorporation, which Sir William Diamond favours, because it will remove overlapping and the treasurer's personal responsibility for the overdraft. At the moment the board of management numbers 600, an egregious position indeed. It is proposed to form committees for regular contributions in each ward of the city, and to approach every guild and chapel and organisation for similar support. By these means it is hoped to set right the balance sheet, which after all that has been done still showed a deficit of £20,000 on the year, though over £30,000 was at one time feared.

The Beaconsfield War Memorial Committee has accepted a tender from Messrs. George Biggs and Sons, of Kingshire, of £32,315 for the new hospital. Building will begin shortly. The tender is £7,000 less than the lowest offered last year, and from the same firm.

Charities will benefit to the extent of £70,000 under the will of the late Mrs. Elizabeth Clarke, of Newton Abbot, Devon. She left £24,000 to be distributed within six months to such charities as her trustees might determine apart from £1,000 each to Newton Abbot Hospital and many other institutions.

Though seven of the general wards at King's College Hospital were closed for half the year during 1921, the sum received from patients' payments was £7,926, compared with £5,643 in 1920, when all the wards were open. The Maternity Ward, reduced from 24 to 12 beds, contributed nearly one quarter of this total, or an average of £2 a week per patient.

At the annual meeting of the Royal London Ophthalmic Hospital it was announced that an anonymous donation of £1,000 had been received for the special fund for an ophthalmic convalescent home. Owing to the critical financial position of the hospital, the donor has allowed the money to be used as a loan to the general fund.

The Sheffield Joint Consultative and Advisory Hospitals' Council records that there are over 55,000 contributors to the 1d. in the £ scheme there. Contributors when ill are exempt from patients' payments at any of the five hospitals. All the City police are subscribers to the scheme, which at present yields £30,000 a year. Mr. Lamb is the secretary.

The subscribers to the Malvern Hospital have agreed to the sale of the building known as the Old Rural Hospital, Newtown, to the Malvern Boys' Club for £1,000. Should the Club ever get into financial difficulties, any assets after the payment of the Club's debts will revert to the Malvern Hospital. The sale of the building has been discussed for years.

At the annual general meeting of the Governors of St. Mark's Hospital for Cancer, Fistula and other diseases of the Rectum, it was stated that the purchase of the adjoining site had been completed, and that £10,000 was needed to build a much-needed extension of the premises. At present many sufferers from the painful diseases treated by the hospital have to wait as long as three months for admission.

The opening of the Howard de Walden Institute as an extension of the West Kent General Hospital, Maidstone, brings its number of beds to over 100. The building was purchased out of the appeal started two and a half years ago which produced £56,700. It was a town and country appeal with active parish committees. Nine beds were endowed by nine villages. Mr. Gerald Mercer and Mr. Edward Hills were the chief organisers.

Royal processions which end at the Abbey generally benefit the funds of Westminster Hospital, which made £17,000 by the sale of seats for King George's Coronation. At the time of writing the hospital does not expect to be so fortunate over Princess Mary's wedding. The Secretary, Capt. Power, M.C., has announced that the lowest tender for building a stand to seat 500 sightseers was £1,000. But we hope that both the Westminster Hospital and St. George's Hospital will do well out of their positions on the processional route.

The workpeople's contributions to Blackburn and East Lancashire Royal Infirmary reached £9,425 last year, despite unemployment. Besides this, the appeal to farmers produced £740, and the promise of nearly as many annual subscribers. This leads the committee to hope that sectional subscriptions on similar lines may be organised among the professional and business classes. A sum of £40,000 has been received toward the proposed war memorial wing, but the efforts to complete the £100,000 originally asked for are in abeyance at present. The Mayor, however, has suggested that district committees in adjacent areas should seize the first favourable moment to raise part of the capital sum.

